# Autoimmune Myasthenia, Primary Adrenal Insufficiency, and Progressive Hypothyroidism Due to Pembrolizumab and Axitinib Combination Regimen

**DOI:** 10.7759/cureus.16933

**Published:** 2021-08-06

**Authors:** Nikole Figueroa-Perez, Rahul Kashyap, Deepinder Bal, Syed Anjum Khan, Vishwanath Pattan

**Affiliations:** 1 Medicine, University of Medicine and Health Sciences, New York City, USA; 2 Internal Medicine/Critical Care, Centennial Medical Center, Hospital Corporation of America (HCA) Healthcare, Nashville, USA; 3 Internal Medicine, Centennial Medical Center, Hospital Corporation of America (HCA) Healthcare, Nashville, USA; 4 Critical Care Medicine, Mayo Clinic Health System, Mankato, USA; 5 Endocrinology, Wyoming Medical Centre, Casper, USA

**Keywords:** myasthenia, adrenal insufficiency, hypothyroidism, thyrotoxicosis, autoimmune, pembrolizumab, axitinib

## Abstract

Immune checkpoint inhibitors (ICI) and tyrosine kinase inhibitors (TKI) have been among the increasingly used antineoplastic agents for advanced cancers including renal cell carcinoma (RCC). Although these antineoplastic agents have broad range of efficacy, rare adverse events - mild and fatal, acute and chronic, immune and non-immune mediated - have been reported. We report a case of a 73-year-old Caucasian male patient with stage IV right-sided clear cell RCC who was treated with a pembrolizumab-axitinib combination regimen and suffered life-threatening, acute onset immune-related myasthenia gravis (MG), subsequently progressive hypothyroidism, and primary adrenal insufficiency.

## Introduction

Pembrolizumab is a humanized IgG4 kappa monoclonal antibody that inhibits programmed cell death 1 receptor (anti-PD1 antibody) which has efficacy in numerous malignancies including renal cell carcinoma (RCC) [1]. Axitinib is a tyrosine kinase inhibitor (TKI) of vascular endothelial growth factor receptors (VEGFR) 1, 2, and 3 [2], which has shown efficacy in RCC in combination with pembrolizumab [3]. Although combining anti-PD1 immune checkpoint inhibitor (ICI) with TKI of the vascular endothelial growth factor (VEGF) pathway has been characterized by excess toxicity, the combination of axitinib plus pembrolizumab was reported to be tolerable [3]. A recent systematic review and network meta-analysis showed that pembrolizumab plus axitinib might be the optimum treatment for intermediate-risk and poor-risk patients with advanced/metastatic RCC [4]. However, these medications are reported to cause adverse reactions, some of which are predictable like hypothyroidism (with axitinib) and others unpredictable like myasthenia gravis (MG) [5]. Pembrolizumab has been reported to cause both primary and secondary adrenal insufficiency (due to hypophysitis), and primary hypothyroidism [6]. In addition, immune-mediated pericarditis, myositis, and myocarditis have also been reported with the use of Pembrolizumab. Axitinib has been reported to cause progressive hypothyroidism due to direct effects on the thyroid gland [7]. In this case report, we discuss autoimmune myasthenia, thyroid, and adrenal perturbations associated with axitinib and/or pembrolizumab with a systematic review of the literature.

## Case presentation

**Figure 1:**
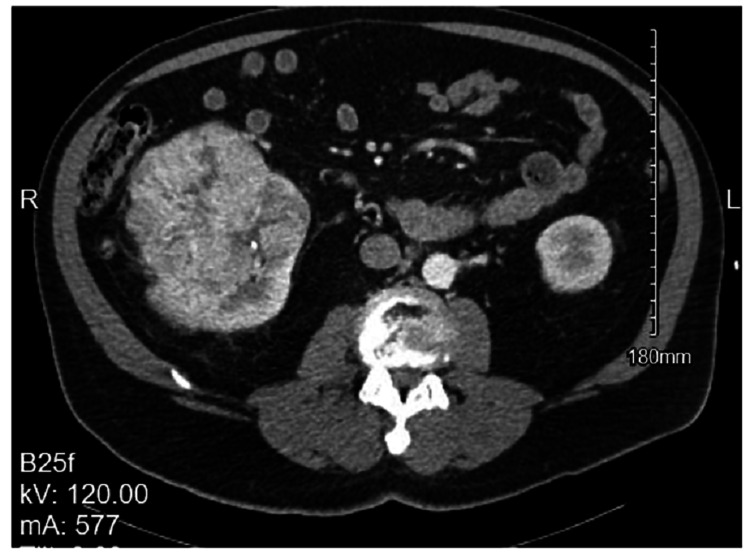
CT abdomen and pelvis with contrast showing right-sided renal cell carcinoma

A 73-year-old Caucasian male with a history of hypothyroidism since 2013 presented with aching pain in the right lower abdomen which was progressively worsening for approximately six weeks. The patient underwent computerized tomography (CT) scan in 2019, revealing a large right renal mass measuring 16.6cm x 13.7cm x 12.4cm (height x width x anteroposterior) without invasion into the renal vein, artery, or inferior vena cava (IVC) (Figure 1). The patient underwent a right radical nephrectomy. Pathology showed a unifocal tumor measuring 11.8cm, Furhman grade-III clear cell carcinoma without sarcomatoid features (pT3a N0 M1, stage IV) with metastases to the lungs.

The patient was started on a regimen consisting of premprolizumab 200mg every three weeks and axitinib 5mg twice daily. Eighteen days after starting pembrolizumab (one dose) and 13 days after starting axitinib, the patient presented to the emergency department due to one-week history of progressive fatigue and shortness of breath. He complained of a level of fatigue he had never experienced before and even had difficulty holding his head up. While in the emergency room he was experiencing rapid shallow breathing, blurred vision, and diplopia. 

On examination, his heart rate was 101 beats per minute, blood pressure 143/107mmHg, respiratory rate 26 breaths per minute, oxygen saturation 91% on 2L of oxygen by nasal cannula, and temperature 36.4°C. On pulmonary examination, mild bilateral basilar crackles and rapid shallow breathing were noted. On neurological examination, the patient had bilateral ptosis and impaired abduction of the eyes bilaterally with diplopia on lateral gaze. His tongue was at midline and palate elevation was slow but symmetric. 

His head and shoulder shrug were four of five, and he had bilateral weakness of the neck flexor muscles three of five. He had normal muscle bulk and tone in all muscle groups of both upper and lower extremities bilaterally. The pronator drift test was performed, and it was positive bilaterally. Strength in both upper and lower extremities was five of five in both flexors and extensors. His deep tendon reflexes were symmetric, the sensation was intact to light touch, and there was no history of ataxia. The patient was started on ceftriaxone and metronidazole to treat aspiration pneumonia. A trial of continuous positive airway pressure (CPAP) failed and unfortunately, the patient had cardiopulmonary arrest with pulseless electrical activity. He received chest compressions for 2 minutes and one dose of epinephrine before the return of spontaneous circulation (ROSC) was achieved. The patient was intubated, placed on ventilator support, and admitted to the intensive care unit (ICU).

Laboratory results were suggestive of a hyperthyroid state with suppressed thyroid-stimulating hormone (TSH) and elevated free tetraiodothyronine (fT4) (Table 1). The patient’s levothyroxine was stopped. A head CT scan was performed and did not show any signs of intracranial bleed. Brain MRI was unremarkable, and a CT angiogram of his chest ruled out pulmonary embolism. The patient was provisionally diagnosed with myasthenia gravis (MG) based on high clinical suspicion. He received three doses of intravenous immunoglobulin (IVIG 65g q24h), 125mg IV methylprednisolone daily, and was also treated with oral pyridostigmine 120mg every 6h. The patient was then started on oral prednisone 60mg daily and the dose was gradually tapered. A laboratory panel for myasthenia gravis showed elevated acetylcholine receptor (ACHR) binding antibodies of 14nmol/L (reference range: 0-0.24 nmol/L), ACHR modulating antibody of 45% (reference range: 0-20%), ACHR blocking antibody of 63% (reference range: 0-25%), muscle-specific kinase (MUSK) antibody undetectable. Interestingly, the patient had a history of mild diplopia for six to seven days, one year prior.

**Table 1:** Serial thyroid laboratory panel IT: immunotherapy; TSH: thyroid-stimulating hormone; T4: tetraiodothyronine or thyroxine

Labs with reference range and time from immunotherapy (IT)	TSH (µIU/mL) reference range 0.465-4.68	Free T4 (ng/dL) reference range 0.78-2.19	Levothyroxine dose (µg per day)	Comment
2 years prior to IT	3.2		50	
18 days after IT	<0.001	2.95	50 (stopped)	The hyperthyroid phase of thyroiditis
9 weeks after IT	43.1		25 (started)	The hypothyroid phase following thyroiditis
16 weeks after IT	9.54	0.63	Increased to 50	
24 weeks after IT	10.3	0.78	Increased to 100 (but patient remained on 50)	
31 weeks after IT	7.04	0.76	Increased to 75	
37 weeks after IT	4.57	0.96	Increased to 125	
47 weeks after IT	2.49	1.37	125	
57 weeks after IT	7.05		Increased to 150	
60 weeks after IT	8.03	0.95	Increased to 175	
64 weeks after IT	1.6	1.23	175	
69 weeks after IT	1.73	1.05	175	

The patient had a history of hypothyroidism diagnosed in 2013, with an elevated TSH of 9.25. He was started on levothyroxine 25µg daily. His TSH was 4.52 after six months, and his levothyroxine dose was increased to 50µg daily. A repeat TSH was 3.49 in 2014. In 2019, before the start of immunotherapy, his TSH was 3.2. Initiation of axitinib and pembrolizumab led to hyperthyroidism due to transient thyroiditis which transitioned into hypothyroidism. Autoimmune workup for thyroid (thyrotropin receptor antibody, thyroperoxidase antibody) was negative.

Although pembrolizumab was discontinued after the first dose due to myasthenic crisis, the patient’s axitinib dose was maintained, and he required progressive increases in his levothyroxine dosage over time. He was also on a tapering dose of prednisone since myasthenia gravis was diagnosed. The patient was on a supraphysiological dose of prednisone for six months, physiological dose of 5mg for three months, and then switched to hydrocortisone 15mg per day for two months, and finally hydrocortisone 10mg daily. After skipping one dose of hydrocortisone, 8 AM labs were drawn and ACTH was elevated to 82pg/mL (7.2-63) with low cortisol of 2.4mcg/dL, and the patient reported severe fatigue, exhaustion, and dizziness due to the skipped dose. An ACTH stimulation test was done without skipping once daily hydrocortisone dose (per patient preference) which showed the following results: baseline cortisol 7.8 mcg/dL (other baseline labs were ACTH 49 pg/mL, dehydroepiandrosterone sulfate (DHEAS) 26mcg/dL {24-244}, renin activity 7.7ng/mL/h {0.6-3.0}, aldosterone 7.7ng/dL {upper limit of normal 21ng/dL}), 30-minute cortisol 12.9 mcg/dL, and 60-minute cortisol 13.9 mcg/dL. Testing for 21-hydroxylase antibody was negative. Prolactin was 9.4ng/mL (6-23) and total testosterone 310ng/dL (240-950). The patient was then placed on a replacement dose of hydrocortisone, 15mg in the morning and 10mg in the afternoon, and fludrocortisone 0.1mg daily.

## Discussion

ICIs and TKIs are increasingly being used as antineoplastic agents for the treatment of advanced RCC. Treatment with axitinib plus pembrolizumab has been reported to be tolerable. However, we report myasthenia gravis, thyrotoxicosis from transient thyroiditis followed by hypothyroidism, and primary adrenal insufficiency as adverse effects from using this combination to treat a patient with clear cell carcinoma.

This patient had a previous history of transient diplopia and hypothyroidism and was being treated with a low dose of levothyroxine 50µg. His symptoms began within 18 days of the combination therapy, presenting with a life-threatening myasthenic crisis and resultant cardiopulmonary arrest. Due to his condition, he required initial resuscitation, hospitalization, intubation, ventilator support, and admission to the ICU. The diagnosis of myasthenia gravis was confirmed with a lab panel that showed elevated acetylcholine receptor binding antibodies, and the patient was treated with prednisone. After his treatment with prednisone was eventually stopped, his ACTH levels were elevated and cortisol was low and the patient had symptoms of fatigue and dizziness. Adrenal insufficiency (AI) was demonstrated by an ACTH stimulation test. The patient was treated with hydrocortisone and fludrocortisone replacement which improved the symptoms.

Pembrolizumab is a monoclonal antibody that acts as an immune checkpoint inhibitor against programmed-cell- death-1 receptor in T cells. The literature data on the development of hypothyroidism, adrenal insufficiency, and myasthenia gravis with the use of axitinib and pembrolizumab is summarized in Table 2 [5-14]. In our patient, symptoms of MG began within 18 days of initiation of treatment. The timing of the symptoms in the comparing cases ranged from one to six weeks after initiation of pembrolizumab. As in our patient, hospitalization and ventilator support requirements have also been reported as complications. MG triggered by ICIs is more likely to be severe and life-threatening on presentation than the sporadic MG with reported mortality rates ranging from 20% to 28.6% and approximately 50% requiring mechanical ventilation [14-16]. In the study by Haugh et al., it was noted that in some cases, symptoms can be due to exacerbation of known myasthenia gravis [14]. Our patient had symptoms of mild diplopia one year prior, so it may be possible that Pembrolizumab exacerbated latent autoimmune myasthenia. 

**Table 2:** A literature review on the development of hypothyroidism, adrenal insufficiency, and myasthenia gravis with the use of axitinib and pembrolizumab ACTH: adrenocorticotropic hormone; MG: myasthenia gravis; N/A: not applicable; TSH: thyroid-stimulating hormone; ULN: upper limit of normal

Studies	Drug	Rate of hypothyroidism	Incidence of hypothyroidism	Rate of myasthenia gravis	Rate of adrenal insufficiency	Notes
Paepegaey et al., 2017 [6]	Pembrolizumab	Thyrotoxicosis - 5 months. Hypothyroidism- 5.5 months	N/A	N/A	1 month after discontinuation of the drug (used for 9 months)	Adverse effects may occur after discontinuation of the treatment
Fujiwara et al., 2012 [7]	Axitinib	The prominent increase in TSH seen between days 200-300 from initiation (compare to our case btw 6-12 months)	N=18; 16 (89%)	N/A	N/A	Thyroid hormone replacement therapy was given to patients to prevent grade 3/4 fatigue
Daimon et al.,2012 [8]	Axitinib	Average: 2. 83 weeks (weeks 1- 5)	N=21; 17 (81%)	N/A	N/A	4 patients developed destructive thyroiditis-induced thyrotoxicosis. The study indicates that prior hypothyroidism was a risk factor for thyrotoxicosis upon treatment
Mukohara et al., 2010 [9]	Axitinib	Within 29 days of treatment	N=12; 11 (92%)	N/A	N/A	In this study, decreased TSH developed in 3 patients (25%) for 5-8 weeks, followed by an increase in TSH above ULN. TSH changes and fatigue onset appeared to correlate
Bekki et al.,2020 [10]	Pembrolizumab	N/A	N/A	N/A	Isolated ACTH deficiency- after 7 weeks of the first dose (2 cycles of Pembrolizumab)	Severe fatigue was the main symptom. The patient was hospitalized for 13 days and was discharged after her fatigue was reduced with hydrocortisone therapy
Robert et al.,2015 [11]	Pembrolizumab	N/A	Group receiving Pembrolizumab every 2 weeks (N=278); 10.1% (28) Group receiving Pembrolizumab every 3 weeks (N=277); 8.7% (24)	N/A	N/A	Fatigue developed in 20.9% and 19.1% of patients receiving pembrolizumab every 2 weeks and those receiving it every 3 weeks respectively
Tanaka et al., 2020 [12]	Pembrolizumab	N/A	N/A	N/A	Isolated ACTH deficiency developed eight days after the eighth cycle of Pembrolizumab 200mg (every 3 weeks)	Presenting symptoms: generalized fatigue and appetite loss. Hospitalization was required. Treated with Hydrocortisone 15 mg/day. After the patient was discharged, ACTH deficiency persisted
Hibino et al.,2018 [5]	Pembrolizumab	N/A	N/A	Symptoms developed after 2^nd^ infusion (day 38 after initiation of treatment)	N/A	The patient was an 83-year-old male that tested negative for antibodies related to MG (seronegative). The patient continued to have neurological symptoms after oral Pyridostigmine treatment (60 mg, three times daily for seven days). Treated with oral Prednisolone (20mg, once daily) on day 45 with full resolution of neurological symptoms by day 100
Liu et al., 2019 [13]	Pembrolizumab	N/A	N/A	Within 7 days after the second infusion	N/A	The patient was a 73-year-old male. Hospitalization was required. Residual ptosis and persistent abduction defect after 4 days of treatment
Haugh et al., 2020 [14]	Pembrolizumab	N/A	N/A	Median onset of 29 days	N/A	N=65 Most patients required hospitalization. 40-50% required mechanical ventilation. Associated Myositis (in 1/3 of cases) and/or myocarditis (in 8% of patients)- more frequently noted in those with thymoma. Fatality in up to 20% of cases

The patient was treated with a tapering dose of prednisone since his initial presentation and diagnosis of MG. AI was diagnosed after his treatment with prednisone was tapered and stopped. Due to his treatment, the time of onset for AI could not be assessed. The patient was on a supraphysiological dose of prednisone for six months, physiological dose of 5mg for three months, and then switched to hydrocortisone 15mg per day for two months, and, finally, hydrocortisone 10mg daily before the patient was tested for AI. AI is a known adverse effect of the use of Pembrolizumab. In some cases, the onset of AI has occurred even after pembrolizumab treatment has been discontinued (Table 2). Fatigue is a common main symptom and hospitalization is often required. It has been reported that corticosteroids help reduce fatigue.

Furthermore, thyrotoxicosis followed by hypothyroidism has been reported in the literature as another complication of pembrolizumab, occurring within five months of treatment initiation (Table 2). This is in concordance with our patient presentation as he developed symptoms of thyroiditis within approximately one month. He experienced a hyperthyroid phase of thyroiditis that began 18 days after therapy with pembrolizumab, during which his levothyroxine 50µg was discontinued. Nine weeks after pembrolizumab therapy, the patient became hypothyroid and was treated with levothyroxine 25µg. Pembrolizumab was discontinued but axitinib was maintained. No autoimmune etiology was found and testing for thyroperoxidase and thyrotropin receptor antibodies were negative.

Cases of life-threatening immune-related cardiotoxicities have been reported as complications of using pembrolizumab. Zarifa et al. presented the incidence of cardiovascular toxicities with the use of multiple ICIs and they noted that pembrolizumab caused myocarditis within 15 weeks and cardiac arrest within 20 weeks of initiating treatment in a study population of 834 patients [17]. Heinzerling et al. reported a case of a metastatic melanoma patient that suffered from cardiac arrest after his ninth infusion with pembrolizumab [18]. He had to be defibrillated and as in our patient, intubated and placed in the ICU. Recovery after high-dose systemic corticosteroids was reported. Katsume et al. reported a complete atrioventricular block with wide QRS complexes in a patient treated with pembrolizumab [19]. This patient did not have any previous cardiovascular disease and was diagnosed with pembrolizumab-induced myocarditis within 16 days of his first dose.

Axitinib is a TKI of VEGFRs 1, 2, and 3. Hypothyroidism has been reported as a complication of the medication’s effect on the thyroid gland. For this reason, the patient’s thyroid hormone levels were frequently monitored. As previously mentioned, he had a prior history of hypothyroidism. After pembrolizumab was discontinued, the patient required progressive increases in his levothyroxine dosages during the following year. His requirements increased up to 175µg of levothyroxine by 60 weeks after the immunotherapy initiation. The case reported by Daimon et al. indicates that prior hypothyroidism is likely to be a risk factor for thyrotoxicosis [8]. In other case reports, the onset of hypothyroidism has occurred within the first month of axitinib treatment with fatigue being a prominent symptom (Table 2). Although our patient did not experience these complications, immune-mediated pericarditis, myositis and myocarditis have also been reported in the literature with the use of pembrolizumab (Table 2).

## Conclusions

The patients being treated with axitinib and pembrolizumab should have regular screening and monitoring of their thyroid hormone and cortisol levels. Since myasthenia gravis may be exacerbated by pembrolizumab, it is important to check for acetylcholine receptor binding antibodies and be aware of any previous myasthenia gravis symptoms. Although our patient did not experience these complications, immune-mediated pericarditis, myositis and myocarditis have also been reported with the use of pembrolizumab, so close cardiac monitoring before, during, and after treatment is crucial. In conclusion, when treating cancers with pembrolizumab and axitinib, it would be ideal to consult a multidisciplinary team of specialists, including oncologists, endocrinologists, and cardiologists contributing to the care of these patients. 
